# Primary Cutaneous B-Cell Lymphoma Imitating Pyoderma Gangrenosum: A Rare and Complex Diagnostic Challenge

**DOI:** 10.3390/jcm15031138

**Published:** 2026-02-02

**Authors:** Maria Markowska, Łukasz Chętko, Natalia Bień, Maria Rajczak, Magdalena Ciążyńska, Joanna Narbutt, Aleksandra Lesiak

**Affiliations:** 1 Department of Infectious, Tropical and Parasitic Diseases for Children, Władysław Biegański Voivodeship Specialist Hospital, Gen. Karola Kniaziewicza Street 1/5, 91-347 Lodz, Poland; maria.k.markowska@gmail.com; 2 Student Scientific Research Club of Experimental, Clinical and Procedural Dermatology, Medical University of Lodz, Pomorska Street 251, 92-213 Lodz, Poland; lukasz.chetko@stud.umed.lodz.pl; 3 International Doctoral School, Medical University of Lodz, Hallera 1 Square, 90-647 Lodz, Poland; natalia.bien@umed.lodz.pl (N.B.); maria.rajczak@umed.lodz.pl (M.R.); 4 Department of Dermatology, Pediatric Dermatology and Dermatological Oncology, Medical University of Lodz, Pomorska Street 251, 92-213 Lodz, Poland; ciazynska.magdalena@gmail.com (M.C.); joanna.narbutt@umed.lodz.pl (J.N.); 5 Chemotherapy Sub-Department and One-Day Chemotherapy Department, Specialist Oncological Hospital NU-MED sp. z o. o., 97-200 Tomaszów Mazowiecki, Poland; 6 Laboratory of Autoinflammatory, Genetic and Rare Skin Disorders, Medical University of Lodz, Pomorska Street 251, 92-213 Lodz, Poland

**Keywords:** primary cutaneous B-cell lymphoma, anaplastic diffuse large B-cell lymphoma, pyoderma gangrenosum mimic

## Abstract

**Background:** Primary cutaneous B-cell lymphomas (CBCLs) are a rare and heterogeneous group of lymphomas, among which the anaplastic variant of diffuse large B-cell lymphoma (A-DLBCL) represents an exceptionally rare entity. Although they typically present as painless and non-ulcerated skin lesions, rare variants may exhibit atypical clinical features. Pyoderma gangrenosum (PG) is a rare inflammatory ulcerative disease that may overlap clinically with other ulcerative dermatoses and pose diagnostic challenges due to the absence of standardized differential algorithms. **Methods:** An 85-year-old male presented with multiple rapidly progressive, painful, ulcerative lesions, initially misdiagnosed as PG and treated with oral cyclosporine with no clinical response. Skin biopsy specimens underwent detailed histopathological and immunohistochemical evaluation. **Results:** The analyses revealed dense infiltration of atypical large lymphoid cells, with CD20, CD45, and CD30 positivity, and a Ki-67 proliferation index of approximately 90%, consistent with primary cutaneous A-DLBCL. Owing to the delayed correct diagnosis, the patient’s condition deteriorated rapidly, leading to his death before appropriate therapy could be initiated. **Conclusions:** The case documents an exceptionally rare cutaneous presentation of A-DLBCL, expanding the extremely limited literature on this enigmatic entity. Furthermore, it underscores the fundamental role of early skin biopsy in the differential diagnosis of non-specific ulcerative lesions, which is critical for ensuring appropriate treatment administration within the therapeutic window in cases of malignancy.

## 1. Introduction

Pyoderma gangrenosum (PG) is a rare inflammatory condition characterized by rapid progression and uncertain etiology [1]. It typically manifests as deep ulcerative lesions with well-defined borders, predominantly occurring on the legs, with pain being the primary symptom [2]. A wide array of conditions, including malignancies, infections, and vascular diseases, frequently mimic the clinical presentation of PG, accounting for up to 30% of misdiagnosed lower-extremity ulcers [3,4,5]. In the absence of standardized diagnostic algorithms, this high rate of clinical overlap significantly increases the risk of misdiagnosis and delays in correct treatment initiation [3].

Primary cutaneous lymphomas are a heterogenous group of lymphoproliferative disorders that primarily involve and remain confined to the skin at the time of diagnosis. The diagnosis of cutaneous B-cell lymphoma (CBCL) is established through the analysis of a skin biopsy specimen and immunohistochemical examination [6,7]. CBCLs are rare, accounting for approximately 20% of all primary cutaneous lymphomas. They generally have a favorable prognosis and are typically slow-growing [7,8]. Anaplastic diffuse large B-cell lymphoma (A-DLBCL) is an exceptionally rare morphologic variant, constituting less than 3.4% of all DLBCL cases [9,10]. Cutaneous involvement in A-DLBCL is remarkably uncommon, as the disease is predominantly nodal, making its skin-only presentation a significant clinical rarity [11]. Although morphologically defined in the 2017 World Health Organization classification, it remains an enigmatic entity within the spectrum of lymphoid malignancies, which only a limited number of studies have addressed so far [10].

This case report documents an exceptionally rare cutaneous presentation of the anaplastic variant of A-DLBCL, which followed a fatal course owing to initial misdiagnosis. Therefore, it also aims to enhance clinical awareness in the diagnostic process of pyoderma gangrenosum (PG) and emphasize the critical role of histopathological examination in the differential diagnosis of ulcerative dermatoses [1].

## 2. Case Presentation

**Figure 1:**
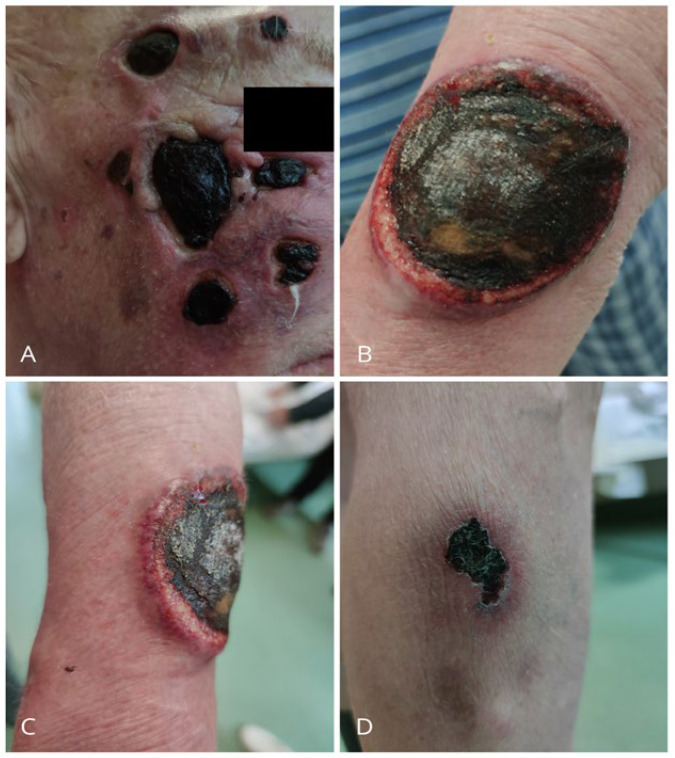
Patient clinical presentation. (**A**) Multiple ulcerated skin tumors of A-DLBCL with necrotic crust located on the face. (**B**,**C**) Round, ulcerated lesion of A-DLBCL with undermined, reddish-violaceous wound margins, peripheral erythema, and necrotic crust located on the forearm. (**D**) Lesion of A-DLBCL located on the lower leg.

An eighty-five-year-old Caucasian male presented to the Dermatology Department on June 8th with multiple painful (VAS 5) ulcerations and necrotic crusts on the face, left forearm, right lower leg, and thigh (Figure 1A–D). The lesions had developed progressively over the previous three months and exhibited reddish-violaceous borders, peripheral erythema, and undermined wound margins, without any preceding injury. The patient reported rapid ulcerative evolution of the lesions that initially presented as erythematous papules and/or patches with the most recent one appearing on the lower leg (Figure 1D). Medical history included atrial fibrillation, chronic heart failure (CHF), hypothyroidism, hypertension, and benign prostatic hyperplasia, as well as a previous pacemaker implantation. There was no history of autoimmune diseases or malignancy. 

Upon admission, the patient was fully conscious, oriented, and hemodynamically stable, with vital signs as follows: arterial blood pressure of 100/60 mmHg, heart rate of 70 bpm, body temperature of 36.7 °C, and respiratory rate of 22 breaths per minute.

The clinical presentation was initially consistent with pyoderma gangrenosum (PG). Consequently, oral ciclosporin A (5 mg/kg body-weight/day) was administered following the initial assessment.

Laboratory tests performed within the following days showed a normal white blood cell count and elevated C-reactive protein level (64.88 mg/L), respiratory alkalosis (pH = 7.470, pCO_2_ = 29 mmHg, pO_2_ = 45 mmHg, HCO_3_^−^ = 21.1 mmol/L), and transient hyponatremia (130 mmol/L) and hypokalemia (3.28 mmol/L). Additional findings included bilateral pleural effusion, attributed to concomitant CHF exacerbation due to no other abnormalities being observed on chest X-Ray and CT-scan. Urine culture was positive for *Enterococcus faecalis*, whereupon targeted antibiotic therapy was initiated.

On June 15th, a triangular incisional biopsy (12 × 4 mm) was performed on the left forearm under local infiltration anesthesia with 1% lidocaine. The specimen was oriented with the base at the wound margin (including healthy skin) and the apex extending toward the center to encompass the necrotic zone, ensuring a cross-section through all macroscopic areas. The excision included the dermis and superficial subcutaneous fat. Hemostasis was achieved with a hemostatic suture, followed by application of a sterile dressing with povidone–iodine (Betadine). After the procedure, an exaggerated skin reaction with aggravated erythema, edema, and extension of the ulceration beyond the biopsy site occurred within the following days.

Further laboratory tests revealed an increase in NT-proBNP (from 5453 to 11,577 pg/mL) and D-dimer (553 to 893 mcg/L) levels, as well as elevated serum LDH (445 U/I) and decreased serum albumin (2.03 g/dL) levels, with no tumor markers detected. The patient’s condition deteriorated rapidly, necessitating his transfer to the Internal Medicine Department on 24 June and leading to his death before the biopsy results were obtained.

**Figure 2:**
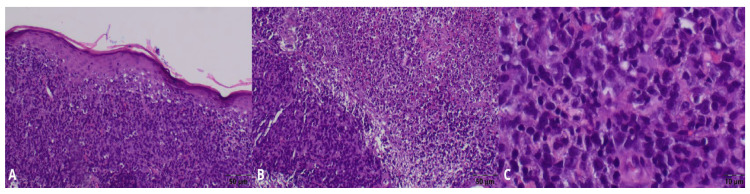
Histopathological examination. (**A**–**C**) Biopsy of the lesions—diffuse infiltrate of atypical lymphoid cells filling the dermis. Numerous mitotic figures.

**Figure 3:**
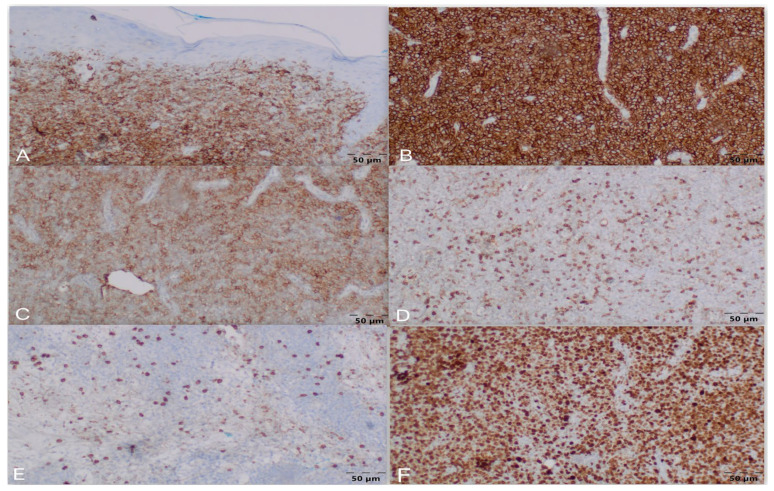
Immunopathology. Microscopic image corresponding to primary cutaneous diffuse large B-cell lymphoma, anaplastic variant. Neoplastic lymphocytes: positive: (**A**) CD45 (lymphoid cells), (**B**) CD20 (B-cells), and (**C**) CD30 (positive in large neoplastic cells, consistent with anaplastic variant); negative: (**D**) CD4 and (**E**) CD8 (highlight reactive T-cells); (**F**) Given Ki-67 = 90%.

Histopathological examination of the biopsy received on June 30 revealed dense infiltration by atypical large lymphoid cells, inconsistent with PG and suggestive of a cutaneous lymphoma instead. (Figure 2A–C) Immunohistochemistry showed CD45, CD20, and CD30 positivity in the tumor cells, with CD4 and CD8 staining limited to reactive T-cells, and was negative for CK PAN. The Ki-67 proliferation index was approximately 90%. Based on these findings, the diagnosis of primary cutaneous diffuse large B-cell lymphoma, anaplastic variant (A-DLBCL), was formed. (Figure 3A–F). The rapid terminal course of the disease precluded further tests necessary for its complete staging and characterization, as well as appropriate treatment administration.

## 3. Discussion

The classification and differential diagnosis of primary cutaneous diffuse large B-cell lymphomas have been extensively discussed and remain problematic. The anaplastic variant of DLBCL was first defined in the 2017 WHO classification. Previously, the 2005 WHO-EORTC classification recognized two types: primary cutaneous diffuse large B-cell lymphoma, leg type (PCDLBCL-LT), and primary cutaneous follicle center lymphoma (PCFCL) with a diffuse growth pattern. Rare DLBCL cases initially presenting in the skin that could not be classified as either of the above were previously grouped as “PCDLBCL, other”; however, this category was excluded from the 2018 update of the WHO-EORTC classification due to interpretative difficulties. In rare cases that cannot be classified as PCDLBCL-LT or PCFCL, a diagnosis of primary cutaneous DLBCL-not otherwise specified (DLBCL-NOS) should be made. This contributes to the limited research on different variants of PCDLBCLs [8,12]. 

Although CBCLs usually present as painless, non-ulcerated solitary or multiple nodules or tumors, with firm consistency, and patches or plaques, rare variants can exhibit atypical clinical features [7,8]. In contrast to the typically indolent and painless course of most CBCLs [1,7], our patient exhibited a rapidly progressive and painful ulcerative presentation. Such clinical presentation of A-DLBCL is exceedingly rare, with only a few isolated case reports documenting similar manifestations available in the literature to date.

Asano et al. reported a rare case of A-DLBCL with both nodal and cutaneous lesions. A 67-year-old man presented with cervical lymph node swelling and tenderness. Following biopsy of the lymph node, a large, round ulceration developed on the skin, with undermined edges and peripheral erythema clinically mimicking PG. Biopsy confirmed the anaplastic variant of DLBCL [11]. The clinical appearance of the lesion as similar to PG suggested a possible connection to cutaneous trauma. Of note, in our patient’s case, the biopsy aggravated the already existing cutaneous lesions. This phenomenon, although observed in PG, may also be linked to cutaneous presentations of A-DLBCL if more cases are reported. It is also noteworthy that the patient described achieved complete remission with CHOP chemotherapy, underscoring the importance of correct diagnosis. 

Sica et al. presented a case of an extensively ulcerated lesion on the back of a 72-year-old woman. The lesion, covered by fibrin and displaying peripheral erythema and undermined edges, developed over several weeks. Biopsy revealed primary cutaneous DLBCL, not otherwise specified (DLBCL-NOS), inconsistent with leg-type DLBCL. The authors reported that excellent disease control was achieved following chemotherapy [13].

Both cases exhibited clinical features resembling PG. However, neither affected the lower extremities, the typical site for PG, and both lesions were solitary. Biopsy results were critical in establishing the correct diagnosis, which directly guided the initiation of chemotherapy and led to subsequent clinical improvement.

In contrast, Oguz et al. reported a case of non-anaplastic primary cutaneous CD30(+) large B-cell lymphoma in a 72-year-old woman, presenting as multiple, firm, reddish nodules and slightly infiltrated erythematous plaques, which developed on her upper back over a year. This suggests that the anaplastic variant may be more likely associated with ulceration [14]. More reports are needed to categorize its features accurately.

In 2017, Li et al. [15] published the largest multicentric study on A-DLBCL, including 35 patients over 12 years. Twelve patients (46%) had two or more extranodal sites involved, including the central nervous system, abdomen, gastrointestinal tract, liver, spleen, pancreas, chest wall, thyroid gland, lung, prostate gland, skin, bone, or soft tissue. However, the authors did not specify the type of cutaneous presentation [15].

Notably, some of the similar cases cited report satisfactory disease control. The chemotherapy regimens typically administered to an 80-year-old patient with a diagnosis similar to ours are associated with 2-year overall survival (OS) ranging from 42 to 66% [16,17,18,19,20,21,22] (see: Supplementary File S1). In our patient, the differential diagnosis posed a significant challenge due to both the atypical A-DLBCL features and clinical presentation initially overlapping with that of PG. Considering the strong B-cell immunophenotype, CD30 expression, high proliferation, and aggressive cutaneous presentation, the most fitting diagnosis was anaplastic variant of DLBCL-NOS. Alternative cutaneous lymphoproliferative disorders were excluded based on immunophenotype, EBV status, and clinical features. Thorough differential diagnosis, including other CD30-positive lymphoproliferative disorders, is summarized in Supplementary File S2 [23,24,25,26,27,28,29].

**Figure 4:**
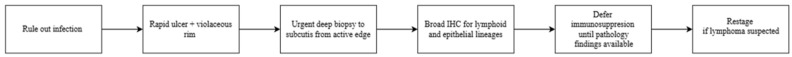
Proposed diagnostic approach to ulcerative dermatoses.

Expected outcomes for anaplastic variant DLBCL-NOS in octogenarians differ from those of primary cutaneous diffuse large B-cell lymphoma, leg type (PCDLBCL-LT), and primary cutaneous follicle center lymphoma (PCFCL), with generally more aggressive behavior and poorer prognosis compared to PCFCL but variable outcomes versus PCDLBCL-LT [8]. Accurate diagnosis is therefore crucial, as it guides appropriate therapy selection and informs prognosis, ensuring patients receive optimal treatment tailored to their specific lymphoma subtype. The results of skin biopsy examination are fundamental in the decision-making process, which is why we propose the diagnostic approach outlined in Figure 4.

Currently, PG remains a diagnosis of exclusion, relying on clinical presentation, patient medical history, and histopathological examination. The condition may at times coexist with underlying systemic diseases or malignancies [3,30]. Immunosuppressive medications used in PG treatment can not only delay the correct diagnosis and appropriate therapy initiation, but also exacerbate underlying conditions such as infections or neoplasms [1,31]. The latter most commonly include myelodysplastic syndromes and leukemias, in which immunosuppression is more likely to result in adverse outcomes than in the clinical scenario observed in our patient [32]. Although several clinical frameworks to guide diagnosis have been proposed, misdiagnosis of PG remains frequent and therefore contributes to increased patient morbidity and mortality [3]. Weenig et al. studied 95 cases of ulcerative dermatoses initially misdiagnosed as PG, highlighting this diagnostic challenge [31].

In recent years, diagnostic tools such as the PARACELSUS scoring system (Table 1) and the Delphi Consensus of International Experts (Table 2) have been proposed to improve diagnostic accuracy [33,34]. Using the PARACELSUS system, the patient in our case met two major (i.e., progressive disease and reddish-violaceous wound margin), three minor (i.e., extreme pain, bizarre wound shape and localized pathergy phenomenon), and one additional criterion (undermined wound border), for a total score of 13 points—exceeding the minimum threshold of 10 points required for a PG diagnosis. The Delphi Consensus, which provides guidelines for PG diagnosis, emphasizes the crucial role of histopathological examination. Analysis of its proposed criteria identified four of the eight minor criteria that maximized diagnostic discrimination in PG cases, achieving 86% sensitivity and 90% specificity [34]. In the present case, the patient was interpreted to meet four minor criteria corresponding to the clinical features of the lesions (i.e., multiple ulcerations—at least one located on the anterior lower leg; peripheral erythema, undermining border, tenderness at the ulceration site; pathergy; history of a papule ulcerating within four days of appearing). Additionally, infection was deemed unlikely due to the lack of evident systemic symptoms or laboratory findings strongly suggestive of infection. Of note, the exaggerated skin reaction, interpreted as pathergy following the biopsy, may as well have resulted solely from proinflammatory cytokine release due to tumor lysis and is by no means pathognomonic for PG. Furthermore, the patient was unable to specify the exact time from lesion onset to ulceration, and, apart from the progression of existing ulcerations, no new “precursor” lesions were observed during the short hospital stay. Nevertheless, the case exemplifies the challenges and limitations of relying solely on clinical presentation for PG diagnosis, which might be misleading, notwithstanding the use of designated criteria.

Recently, AI-based image-analysis and clinical decision support systems have begun to show promise in dermatology workflows, including ulcerative dermatoses [35,36]. In the future, AI-enabled decision-support platforms may also help reduce ‘diagnosis-by-pattern’ error in ulcerative dermatoses such as PG. Such systems could integrate serial ulcer photographs (±dermoscopy) together with structured clinical inputs, including pain severity, temporal progression, lesion distribution, systemic symptoms, and immunosuppression exposure, to generate a standardized differential diagnosis. Importantly, these tools could flag “red-alert” features suggestive of malignancy or atypical disease and prompt early biopsy with immunohistochemistry before initiating high-dose immunosuppression. Embedded alerts within the electronic health record may also support a minimum safety bundle in presumed PG, including deep-edge biopsy, tissue culture, and early dermatopathology review when lesions are multifocal, atypical, or rapidly progressive. Although these systems are not yet routinely implemented, they highlight the potential for AI to assist clinicians in detecting high-risk presentations at an earlier stage and to reduce delays in the diagnosis of serious conditions, including cutaneous lymphoma.

There exist several limitations to this report that merit attention. Firstly, it describes a single case, which limits the generalizability of the findings. Owing to the rapid terminal course of the disease, its further and more detailed characterization was not feasible. Extended immunohistochemical profiling (including PAX5, BCL6, MUM1, BCL2, MYC, FOXP1, GCET1, and CD21/23), EBER-ISH, and FISH analyses for MYC, BCL2, and BCL6 rearrangements were not performed, and therefore, determining the cell of origin according to the Hans algorithm was not possible. Furthermore, the patient’s death prevented subsequent imaging studies, including additional CT scans or PET CT, as well as bone marrow biopsy and serum β2-microglobulin level assessment. Consequently, complete disease staging could not be performed. Given that the patient passed away before appropriate treatment initiation, it was also impossible to demonstrate therapeutic response and evaluate its consistency with the existing literature data.

## 4. Conclusions

Rapidly progressive ulcerative lesions meeting the clinical criteria for PG may, in fact, represent an underlying A-DLBCL, making prompt biopsy prior to immunosuppressive therapy decisive. The case describes an exceptionally rare cutaneous manifestation of the anaplastic variant of A-DLBCL, contributing to the limited body of literature on this enigmatic entity. Further research is necessary to not only better characterize rare CBCL variants, but also to establish standardized approaches for differentiating ulcerative conditions of various origins, ensuring timely treatment initiation in case of underlying malignancies.

## Figures and Tables

**Table 1:** The PARACELSUS score—a diagnostic tool for pyoderma gangrenosum.

Category	Criterion	Score
Major criteria	Progressive disease Absence of differential diagnoses Reddish-violaceous wound margin	3 points each
Minor criteria	Alleviation due to immunosuppressive drugs Characteristically irregular (bizarre) wound shape Extreme pain (VAS > 4) Localized pathergy phenomenon	2 points each
Additional criteria	Suppurative inflammation in histopathology Undermined wound border Systemic disease associated	1 point each

**Table 2:** Diagnostic criteria of ulcerative pyoderma gangrenosum according to the Delphi consensus of international experts.

Category	Criterion
Major criteria	Biopsy of ulcer edge demonstrating neutrophilic infiltrate
Minor criteria	Exclusion of infection Pathergy History of inflammatory bowel disease or inflammatory arthritis History of papule, pustule, or vesicle ulcerating within 4 days of appearing Peripheral erythema, undermining border, and tenderness at ulceration site Multiple ulcerations, at least 1 on anterior lower leg Cribriform or “wrinkled paper” scar(s) at healed ulcer sites Decreased ulcer size within 1 month of initiating immunosuppressive medication(s)

## Data Availability

Data sharing does not apply to this article, as no datasets were generated. Further information regarding the patient’s medical history and clinical management is available upon request from the corresponding author due to privacy considerations.

## Supplementary Materials

The following supporting information can be downloaded at: https://www.mdpi.com/article/10.3390/jcm15031138/s1, Supplement S1 First-line treatment options for an elderly patient (>80 years of age) diagnosed with PCDLBCL-NOS (anaplastic variant); Supplement S2. Example differential diagnosis of a cutaneous neoplasm.

## Abbreviations

The following abbreviations are used in this manuscript:

PG	Pyoderma Gangrenosum
CBCL	Cutaneous B-cell Lymphoma
DLBCL	Diffuse Large B-cell Lymphoma
A-DLBCL	Anaplastic Diffuse Large B-cell Lymphoma
PCDLBCL-LT	Primary Cutaneous Diffuse Large B-cell Lymphoma–Leg Type
PCFCL	Primary Cutaneous Follicle Center Lymphoma
DLBCL-NOS	Diffuse Large B-cell Lymphoma–Not Otherwise Specified
