# Supplementary material for: Primary Cutaneous B-Cell Lymphoma Imitating Pyoderma Gangrenosum: A Rare and Complex Diagnostic Challenge
Source: J Clin Med. 2026 Feb 2;15(3):1138. doi: 10.3390/jcm15031138 (PMC12898250; doi:10.3390/jcm15031138)
Supplement: Supplementary file 1 [file jcm-15-01138-s001.zip › Supplement S1.pdf]

Supplement S1 First-line treatment options for an elderly patient (>80 years of age) diagnosed with PCDLBCL-NOS (anaplastic variant).

Treatment	Clinical rationale	Efficacy
**Chemotherapy regimens**		
R-mini-CHOP	• **standard of care for octogenarians**<br>• attenuated doses of cytotoxic agents<br>• cardiac monitoring required due to doxorubicin	2y OS: 59–66% [16,17]
R-GemOx	• high efficacy with a **favorable cardiac safety profile**<br>• indicated in case of pre-existing cardiac comorbidities	3-year OS: ~65% [16,18]
R-CEOP		non-inferior to R-CHOP [16,19]
R-THP-COP		non-inferior to R-CHOP [16,20]
reduced dose EPOCH-R	• efficacious **alternatives in case of frailty**	non-inferior to R-CHOP [16]
R-COMP		comparable to R-CHOP [16]
R-GCVP	• low intensity regimens, **best for "unfit", frail patients**	2y OS: ~55% [16,21]
Rytuxymab ± RT	• **palliative treatment** if other systemic treatment contraindicated	2y OS: ~42% [22]
Localized **radiotherapy** (RT)	• Add-on to chemotherapy<br>• Primary therapy if systemic treatment contraindicated [7]	

**Supportive care**	
Wound care	Specialized dressings (silver/alginate) and antiseptic cleansing to manage exudate and prevent infection.
Pain control	Systemic opioids and corticosteroids in case of extreme pain.
Metabolic support and tumor lysis syndrome prevention	Hydration, electrolyte correction, allopurynol

OS – overall survival

R-mini-CHOP - Rituximab + reduced doses of Cyclophosphamide, Hydroxydaunorubicin (Doxorubicin), Oncovin (Vincristine), and Prednisolone

R-GemOx - Rituximab + Gemcitabine and Oxaliplatin

R-CEOP - Rituximab + Cyclophosphamide, Etoposide, Oncovin (Vincristine), and Prednisolone.

R-THP-COP - Rituximab + THP-adriamycin (Pirarubicin), Cyclophosphamide, Oncovin (Vincristine), and Prednisolone

EPOCH-R - Etoposide, Prednisolone, Oncovin (Vincristine), Cyclophosphamide, and Hydroxydaunorubicin (Doxorubicin) + Rituximab

R-COMP - Rituximab + Cyclophosphamide, Oncovin (Vincristine), Mitoxantrone, and Prednisolone

R-GCVP - Rituximab + Gemcitabine, Cyclophosphamide, Vincristine, and Prednisolone.
