# Supplementary material for: Primary Cutaneous B-Cell Lymphoma Imitating Pyoderma Gangrenosum: A Rare and Complex Diagnostic Challenge
Source: J Clin Med. 2026 Feb 2;15(3):1138. doi: 10.3390/jcm15031138 (PMC12898250; doi:10.3390/jcm15031138)
Supplement: Supplementary file 1 [file jcm-15-01138-s001.zip › Supplement S2.pdf]

Supplement S2. Example differential diagnosis of a cutaneous neoplasm.

Condition	Morphology	Site of occurence	Immunohisto-chemistry (IHC)	Typical age of onset	Ulceration propensity
**Pyoderma gangrenosum (PG)** [23]	Deep ulcerative lesions with well-defined borders	Predominantly lower extremities	No specific IHC profile; Polymorphous inflammatory neutrophilic infiltrate in histopathological findings	Most common in adults between 40 and 60 years of age, although cases reported across all age groups	Very high
**Primary cutaneous DLBCL, leg type (PCDLBCL- leg type)** [24]	Red-violaceous plaques, nodules or tumors	Predominantly lower extremities	CD20 +<br>CD79a +<br>BCL2 + (90%)<br>IFR4/MUM + (85%)<br>FOXP1 + (90%)<br>MYC + (up to 80%)<br>BCL6 + (60%)<br>p63 + (41-70%)<br>CD10 -<br>High Ki-67	Elderly patients (median ~70-76 years of age), more common in women	Moderate to high (ulceration not rare in bulky or advanced lesions)
**Primary cutaneous follicle centre lymphoma-diffuse growth pattern (PCFCL diffuse)** [25]	Single, raised nodule or plaque. Occasionally a localized cluster of lesions	Mainly head or trunk	CD20 +<br>CD79a +<br>BCL6 +<br>CD10 - in diffuse pattern<br>BCL2 negative to weak positive<br>Ki-67 >30%	Middle-aged adults (~50-60 years of age)	Low- ulceration is uncommon
**Diffuse Large B-Cell Lymphoma, Not Otherwise Specified (DLBCL-NOS)** [26]	Often firm nodules/tumors with rapid growth	Lymph nodes (71%)<br>Extranodal presentation most commonly in gastrointestinal tract, cutaneous presentations are possible too	positive B cell markers (CD20, PAX5, CD79a, CD19, CD22)<br>Cell of origin subtyping: CD10 (30-50%), MUM1 (35-65%), BCL6 (60-90%) CD30+ (14%) associated with anaplastic morphology<br>High Ki67	Median age 70 years but occurs across all age groups	Moderate-high, especially in large necrotic lesions
**EBV positive Diffuse Large B-Cell Lymphoma (EBV+ DLBCL)** [27]	Solitary or multiple firm nodules or tumors	Predominantly extranodal<br>Most common sites in skin (mostly lower extremities, especially the lower legs), lung, tonsil, GI tract	In situ hybridization for EBV encoded small RNA must be positive.<br>CD19+<br>CD20+<br>CD79a+<br>PAX5+<br>CD30 variable<br>BCL 6 + (40 %)<br>MUM 1 + (90%)<br>CD10-<br>CD15-<br>EBNA2 (7-36%)<br>LMP1 (>90%)<br>High Ki-67 high	Mostly older patients (> 50 years)	Moderate-high

**Cutaneous Hodgkin-like lesions (CH- like lesions)**	Varied morphology Nodules/plaque	Primarily lower extremities	Reed-Sternberg-like cells, EBV-positive (EBER+) CD30+ CD15+/- (often in classic HL); PAX5 weak+ MUM1+ typically CD45-	Older adults, most commonly over 50 y.o., but can occur in younger patients when immuno-suppressed	Moderate
Lymphomatoid Papulosis (LyP) [28]	Small papules or nodules, can be red, pink, or violaceous	More common in trunk and extremities but can affect any body part	Activated T helper phenotype, CD3 CD4+ CD30+ CD45RO HLA-DR+ CD25,TIA1 and granzyme B positive usually CD8 negative variable loss of pan T cell antigens CD2, CD5, CD7.	Fifth decade of life	Variable
Primary Cutaneous Anaplastic Large Cell Lymphoma (pcALCL) [29]	80% of cases present as a cutaneous localized nodule or papule with or without ulceration, Multifocal lesions in 20% of cases	Most common in lower extremities or head and neck area<br><br>Extracutaneous dissemination (mainly to regional lymph nodes) in 10% of cases	CD30+ in more than 75% of neoplastic cells; Activated CD4 T cell immunophenotype, CD45+ Cytotoxic proteins: granzyme B, TIA1 and perforin positive, CD15+(~40%); variable loss of CD2; CD5, CD7, CD3, CD8 usually negative; EMA, ALK, CD65 negative	Median 55 years of age	Moderate to high
